# Food insecurity: a neglected public health issue requiring multisectoral action

**DOI:** 10.1186/s12916-023-02845-3

**Published:** 2023-04-04

**Authors:** BMC Medicine

**Affiliations:** The Campus, 4 Crinan Street, London, UK

Food insecurity (FI) describes the lack of consistent access to enough food for every person in a household to live an active, healthy life. It is a global issue: according to the UN, 928 million people were severely food insecure already in 2020—an increase of 148 million on the previous year. In 2021, around 30% of the global population—2.3 billion people—were moderately or severely food insecure, and 11.7 percent (923.7 million people) faced severe FI.

Recent events, such as the coronavirus pandemic and increases in the costs of living, have turned a spotlight on the issue of FI. Contemporary media reports often include news about people struggling to feed themselves and their family appropriately. Recent data from the Food Foundation suggests that the number of UK children in food poverty has nearly doubled in the last year to almost 4 million. In the USA, supplements in benefits for those on the federal Supplemental Nutrition Assistance Program (SNAP), increased due to the pandemic, ended in March in 32 states. This has affected over 30 million people, who will now become more vulnerable to food insecurity. This Editorial will discuss the individual and societal burden of FI and explain why solutions require multisectoral action.

FI remains relatively hidden, especially in high-income countries where there is a perception that it is not a big problem. However, studies have identified that FI prevalence in these countries is unexpectedly high, ranging from 8 to 20% of the population. As a comparison, in 2021, countries in sub-Saharan Africa had an FI prevalence (moderate-severe) of around 63%, countries in Southern Asia had an FI prevalence of 40%, and countries in the Caribbean had an FI of 64%.

Whereas there has been a high awareness of FI in lower income countries, often due to famine resulting from food scarcity, there is only recently a growing awareness of FI in higher income countries, for instance, in the UK through higher usages of food banks. For example, Citizens Advice, just one of the organizations that can supply food vouchers to access a food bank, gave out 14,704 vouchers in August 2022, around 6500 more than in August 2021. This is reflective of a trend of a decade-long rise in food bank use that has seen a wider range of the population having to use them, including teachers, social workers, and public sector workers.

Inequality in high-income countries is the main contributing factor for FI rather than a complete scarcity of resources. Indeed, wealthier countries dispose of more than enough food than could be used to feed people who cannot afford to feed themselves. In the UK alone, this amounts to the disposal of an astonishing 10 million tonnes of food each year.

Risk factors for FI in wealthier countries, such as chronic illnesses, disability, or low-income, are often complex and interconnected and can likely only be resolved fully at a societal level. It is especially complicated as risk factors for FI are driven by inequalities themselves, creating a problematic web that is difficult to resolve. Any meaningful interventions should be targeted across multiple sectors.

Quantification of FI is the starting point of its resolution. A global tool to measure food insecurity and quantify severity was created in 2013: the Food Insecurity Experience Scale (FIES). One of the advantages of the FIES tool is that it is an “experience-based” survey that provides a lived experience and can characterize the risk factors and consequences for FI accurately and across global norms. The FIES tool in particular records anxiety about not having enough food to eat in the future. This anxiety is found to be a common starting experience across the spectrum of severity of FI: as food access conditions worsen, respondents compromise on food choices, resulting in a lowering of the quality and diversity of their diet. This stage is part of the commonly observed obesogenic pathway in FI—resulting in a double burden of malnutrition and obesity that has obvious adverse health effects.

As well as an increased risk in diet-related diseases such as diabetes, those experiencing FI are at heightened odds of being diagnosed with multiple chronic conditions, such as mental health disorders, heart disease, chronic pain, and rheumatological conditions. Consequently, adults experiencing FI are more likely to die prematurely: severely food-insecure adults die an average of 9 years earlier than their food-secure counterparts.

Aside from the individual mental and physical health burden, FI puts a strain on national health systems. Data from Canada, for instance, shows that adults experiencing FI are more likely to be admitted for acute care, stay in hospital for longer, and are more likely to be readmitted than those who are food secure. Strikingly, those who are experiencing severe FI are three times more likely to be admitted to acute care for mental health reasons.

At an academic level, what should the priorities for research be? Various research, in preliminary stages, are small scale and look at the effects of, for example, food prescriptions on diet quality and well-being or the effects of social protection interventions on reducing FI. What is lacking is data on how larger scale interventions, such as governmental policies, could affect FI in terms of health outcomes.

Interventions to limit the scale of FI should clearly be a public health priority: not only will this lead to a better quality of life for citizens, but it will also alleviate the burden on health services. However, the unfortunate truth is that many high-income countries have limited government support, tending to rely upon food assistance delivered through the voluntary sector. This is despite the clear negative correlation between social expenditure and use of food banks. Food banks, run by the voluntary sector, have “become secondary extensions of weakened social safety net” [1]. While the availability of food banks is appropriate to provide a *temporary* safety net to those in need, in themselves, they do not provide a pathway out of the cycle of FI. Appropriate public health initiatives and policies at a national level are needed to break this cycle. Given the complexity and interconnectivity between FI, social determinants, and inequalities, this will require cross-sector action.

FI is a societal problem that will require a societal fix. In high-income countries where the availability of food itself is not the causal factor, there should be no excuse for neglecting this issue.

## Data Availability

Not applicable.
